# Prediction model for etiology of fever of unknown origin in children

**DOI:** 10.1007/s00431-025-06277-4

**Published:** 2025-06-19

**Authors:** Pannachet Rienvichit, Butsabong Lerkvaleekul, Nopporn Apiwattanakul, Samart Pakakasama, Sasivimol Rattanasiri, Soamarat Vilaiyuk

**Affiliations:** 1 Department of Pediatrics, Faculty of Medicine Ramathibodi Hospital, Mahidol University, Bangkok, Thailand; 2 Division of Rheumatology, Department of Pediatrics, Faculty of Medicine Ramathibodi Hospital, Mahidol University, Bangkok, Thailand; 3 Division of Infectious Disease, Department of Pediatrics, Faculty of Medicine Ramathibodi Hospital, Mahidol University, Bangkok, Thailand; 4 Division of Hematology and Oncology, Department of Pediatrics, Faculty of Medicine Ramathibodi Hospital, Mahidol University, Bangkok, Thailand; 5 Section for Clinical Epidemiology and Biostatistics, Faculty of Medicine Ramathibodi Hospital, Mahidol University, Bangkok, Thailand

**Keywords:** Prolonged fever, Infection, Autoimmune, Malignancy, Pediatric

## Abstract

Diagnosing fever of unknown origin (FUO) in children remains challenging, particularly in differentiating between infections, autoimmune diseases, and malignancies. We aimed to develop and validate a prediction model for determining the etiology of pediatric FUO. We retrospectively reviewed medical records of children aged 1–18 years with FUO lasting ≥ 7 days from 2007 to 2023. Clinical and laboratory data were collected. The study was conducted in two phases: (1) model development (development cohort) and (2) internal validation (validation cohort). Multinomial logistic regression and predictive margin analyses were used to construct the model, with performance assessed by the area under the Receiver Operating Characteristic curve (AUC). In the development cohort (*n* = 240, median age: 6.4 years, IQR 3.4–11.6), FUO was attributed to infections (32.5%), autoimmune diseases (34.2%), and malignancies (33.3%). Using infections as a reference, arthritis (OR = 32.8, 95%CI 6.5–166.4) and fever > 30 days (OR = 10.3, 95%CI 2.9–35.4) were predictors of autoimmune diseases; while splenomegaly (OR = 5.2, 95%CI 1.8–15.6), lymphadenopathy (OR = 4.2, 95%CI 1.6–11.2), severe anemia (OR = 9.2, 95%CI 2.3–36.9), thrombocytopenia (OR = 10.0, 95%CI 3.3–30.1), and fever > 30 days (OR = 19.4, 95%CI 5.1–73.8) were predictors of malignancies. Coughing was inversely associated with both autoimmune (OR = 0.1, 95%CI 0.1–0.4) and malignancies (OR = 0.1, 95%CI 0.04–0.4). A computerized prediction model was constructed using these parameters. The validation cohort (*n* = 78) demonstrated good discrimination for infection (AUC = 0.82), autoimmune (AUC = 0.88), and malignancies (AUC = 0.83).

*Conclusions*: A prediction model has been developed and validated to assist pediatricians in differentiating the causes of FUO. It demonstrates good performance and supports data-driven decision-making in pediatric FUO.

**Supplementary Information:**

The online version contains supplementary material available at 10.1007/s00431-025-06277-4.

**Table:** 

**What is Known:**
• *Etiologies for fever of unknown origin (FUO) mainly come from infections, autoimmune diseases, and malignancies.*
• *Pediatric FUO is challenging to diagnose due to its complexity; therefore, delays in diagnosis and treatment are common.*
**What is New:**
• *We identified seven parameters, including arthritis, duration of fever, cough, splenomegaly, lymphadenopathy, anemia, and thrombocytopenia, as significant factors contributing to the etiologies of FUO and incorporated them into our prediction model.*
• *The prediction model can serve as an additional tool to assist physicians in identifying the etiologies of FUO in children, which will facilitate earlier diagnoses and reduce unnecessary testing.*

## Introduction

Fever of unknown origin (FUO) in children is a challenging diagnostic and clinical dilemma. Pediatric FUO is characterized by a prolonged febrile illness without an identifiable source after initial investigation. Early and accurate diagnosis is crucial, as timely treatment can significantly reduce morbidity and mortality, particularly in cases of infectious or neoplastic etiologies [1, 2]. The diagnostic challenge of pediatric FUO is amplified by the diversity of its potential causes and the need to balance invasive investigations with judicious management to avoid unnecessary interventions [3].

The etiologies of pediatric FUO vary significantly by region and demographic context. Common causes include infections, autoimmune diseases, and malignancies, although the proportion of each cause varies across geographic locations and healthcare settings [4, 5]. For instance, infections remain the leading cause in many studies, but in high-income countries, autoimmune diseases and undiagnosed cases are increasingly reported [6, 7]. An Egyptian study identified collagen vascular diseases (30.9%) as the most frequent cause of FUO, which was followed by infections (28.2%), malignancies (17.3%), and miscellaneous conditions (19.1%) [8]. Similarly, a Korean study found that while infections remain the most common cause, connective tissue diseases and malignancies are becoming increasingly recognized following advances in diagnostic capabilities [9].

Several studies have emphasized how geographic differences influence the etiologies of FUO. A Taiwanese study using a four-stage diagnostic protocol revealed that infections accounted for 37.6% of FUO cases, followed by malignancies (17.2%) and collagen vascular diseases (14.0%) [5]. Meanwhile, an Italian survey of pediatricians highlighted variability in their definition and management of FUO, reflecting inconsistencies in diagnostic approaches [3]. In the Philippines, FUO was primarily attributed to infections (43.9%), connective tissue diseases (38.6%), and hematologic or oncologic disorders (14%) [10]. These regional variations underscore the importance of localized epidemiological data in refining diagnostic strategies [11].

Despite advancements in diagnostic techniques, a significant proportion of FUO cases remain undiagnosed [12, 13]. The development of novel biomarkers, molecular diagnostics, and advanced imaging modalities has improved diagnostic accuracy, but these advances are still not universally accessible, particularly in settings with limited resources [14].

A significant gap exists in the development of prediction models to improve diagnoses of pediatric FUO based on clinical parameters and initial laboratory investigations. Current approaches often depend on extensive stepwise testing, which can delay diagnosis and raise healthcare costs [1, 12]. This study aimed to address the research gap by developing and validating a prediction model to classify pediatric patients with FUO into infections, autoimmune diseases, and malignancies, based on clinical and laboratory parameters. The model is intended to provide clinicians with a practical tool to enhance the diagnostic approach for identifying the causes of FUO in children.

## Materials and methods

A cross-sectional study was conducted using a retrospective chart review of children and adolescents aged 1 to 18 years who were treated at the Faculty of Medicine, Ramathibodi Hospital in Bangkok, Thailand, between January 2007 and July 2023. This study was approved by the ethics committee of the Faculty of Medicine Ramathibodi Hospital, Mahidol University, in accordance with the 2013 Declaration of Helsinki (MURA2023/650). The requirement for informed consent was waived due to the cross-sectional retrospective study design. This study was conducted in two phases: (1) development of a prediction model using a development cohort and (2) internal validation using a validation cohort. The development cohort included data collected between January 2007 and December 2020, while the validation cohort consisted of consecutive FUO patients seen between January 2021 and July 2023, based on a 3:1 ratio between the two cohorts. Patients in the validation cohort were selected based on meeting the definition of FUO at presentation, not based on the final cause of the fever. Therefore, the model was validated in an independent FUO cohort.

### Participants

We included children and adolescents aged 1 to 18 years who had fever or presented with a documented body temperature exceeding 38 °C for at least 7 days without a known source at the time of hospital admission. All patients underwent initial diagnostic evaluations before being classified as having FUO. The initial workup was guided by clinical presentation and typically included a complete blood count (CBC), blood and urine cultures, chest radiography, relevant serological tests, and tuberculosis testing. A computed tomography scan of the chest and abdomen was performed if the etiology remained unclear after the initial evaluation. Final diagnoses were confirmed using standardized and disease-specific criteria. Infectious diseases required microbiological confirmation, such as culture, polymerase chain reaction, or serological testing. Autoimmune diseases were diagnosed based on established classification criteria, such as ILAR classification criteria for systemic juvenile idiopathic arthritis (SJIA) [15] and the 1997 ACR, 2012 SLICC, or 2019 EULAR/ACR criteria for systemic lupus erythematosus (SLE) [16–18], or supported by serological testing, biopsy or imaging when appropriate for other autoimmune diseases. Malignancies were confirmed by bone marrow aspiration, tissue biopsy, or other histopathological methods. Thus, both the diagnostic workup and final classification followed standardized and validated protocols across all disease categories. Patients with underlying autoimmune disorders, cancer, or primary immunodeficiency, as well as those who were on immunosuppressive medications, were excluded from the study.

### Data collection

Eligible patients were identified through the electronic medical record system using the International Classification of Diseases, Tenth Revision [ICD-10] codes, which code R501 for “persistent fever” alone, and code R509 for “unspecified fever” in combination with ICD-10 codes for specific diseases commonly associated with FUO (Supplemental Table 1). These conditions were selected based on evidence from previously published literature [6] and consideration of prevalent conditions in our regional epidemiological setting. Among the patients identified based on ICD-10 codes, only those presenting with fever lasting ≥ 7 days were included.

Patient records were comprehensively reviewed to obtain demographic data, duration of fever, final diagnosis, comorbidities, and clinical characteristics. The duration of fever (i.e., number of days) was defined as the duration from the first day until the day that the fever subsided. Clinical symptoms included headache, cough, weight loss, and malaise, whereas physical signs included pallor, arthritis, lymphadenopathy, and hepatosplenomegaly. Laboratory data included CBC, liver function tests, lactate dehydrogenase, and inflammatory markers, including erythrocyte sedimentation rate, C-reactive protein, procalcitonin, and serum ferritin. The final FUO diagnoses were sorted into three etiological categories based on infections, autoimmune diseases, or malignancies. Patients with two conditions or who were undiagnosed were excluded. The first laboratory values were collected from initial investigations during the same hospitalization. Any missing parameters were excluded from the study.

### Statistical analysis

The data were analyzed using STATA software (v. 18, StataCorp, College Station, TX, USA). Continuous variables were presented as means and standard deviations or medians and interquartile ranges, while categorical variables were presented as numbers and percentages. The sample size was estimated based on an α error of 0.05 and a β error of 0.2, given the required 78 cases per etiological category. We compared the continuous data between categories using a one-way analysis of variance for normally distributed data or the Kruskal–Wallis test for non-normally distributed data. A χ^2^ or Fisher’s exact test determines significance in binary or categorical data.

We used multinomial logistic regression analysis with three outcome categories: infections, autoimmune diseases, and malignancies. Infections were set as the reference category to calculate an odds ratio (OR) with a 95% confidence interval (CI) and identify predictors associated with the etiologies of pediatric FUO [19]. We then performed univariable analysis to identify potential predictors, where predictors with *p*-values < 0.05 were considered statistically significant. These parameters and those deemed clinically relevant were included in the multivariable analysis using a backward stepwise approach. The linear predictors (LPs) and probabilities of each outcome category were estimated using the following equations. In addition, the model’s predictive power was further validated using the Hausman–McFadden test to evaluate the independence of irrelevant alternatives (IIA) assumption.

$${\text{LP}}_{\text{autoimmune}} =log\left(\frac{P(Y=autoimmune)}{P(Y=infection)}\right)={\beta }_{\text{0,1}}+{\beta }_{\text{1,1}}{x}_{1}+{\beta }_{\text{2,1}}{x}_{2}+\dots +{\beta }_{p,1}{x}_{p}$$

$${\text{LP}}_{\text{malignancy}} =log\left(\frac{P(Y=malignancy)}{P(Y=infection)}\right)={\beta }_{\text{0,2}}+{\beta }_{\text{1,2}}{x}_{1}+{\beta }_{\text{2,2}}{x}_{2}+\dots +{\beta }_{p,2}{x}_{p}$$

$$\text{Probability of infection }= \frac{1}{1+\text{exp}\left({LP}_{autoimmune}\right)+exp\left({LP}_{malignancy}\right)}$$

$$\text{Probability of autoimmune diseases }= \frac{exp\left({LP}_{autoimmune}\right)}{1+\text{exp}\left({LP}_{autoimmune}\right)+exp\left({LP}_{malignancy}\right)}$$

$$\text{Probability of malignancy }=\frac{exp\left({LP}_{malignancy}\right)}{1+\text{exp}\left({LP}_{autoimmune}\right)+exp\left({LP}_{malignancy}\right)}$$

where $\beta$ is the coefficient value and $x$ is the predictor variable.

The discriminant performance of the prediction model was then evaluated using the area under the Receiver-Operating Characteristic curve (AUC).

## Results

From 318 enrolled cases, the development cohort comprised 240 patients, including 78 (32.5%) with infections, 82 (34.2%) with autoimmune diseases, and 80 (33.3%) with malignancies. The validation cohort comprised 78 patients, including 13 (16.7%) with infections, 30 (38.5%) with autoimmune diseases, and 35 (44.9%) with malignancies. There were no significant differences in age, sex, or duration of fever. Most clinical and physical examinations were not statistically different. However, some symptoms, such as cough and malaise, were significantly lower in the validation cohort, whereas fatigue and arthritis were significantly higher in the validation cohort (Supplemental Table 2).

The final FUO diagnoses in the development cohort are presented in Table 1. In the infections category, the most common diagnoses were *Mycobacterium tuberculosis* (23.1%)*,* Epstein–Barr virus (19.2%), and lower respiratory tract infection (19.2%). In the autoimmune diseases category, the most prevalent diagnosis was SJIA (54.8%), followed by SLE (31.7%) and polyarteritis nodosa (4.9%). In the malignancies category, the most common diagnoses were acute lymphoblastic leukemia (71.2%), followed by neuroblastoma (10%) and acute myeloid leukemia (8.8%).

**Table 1:** The final diagnoses of fever of unknown origin in this study

Infection (*n* = 78)	Autoimmune (*n* = 82)	Malignancy (*n* = 80)
*Mycobacterium tuberculosis* infection 18	Systemic juvenile idiopathic arthritis 45	Acute lymphoblastic leukemia 57
Epstein–Barr virus infection 15	Systemic lupus erythematosus 26	Neuroblastoma 8
Lower respiratory tract infection 15	Polyarteritis nodosa 4	Acute myeloid leukemia 7
Infective endocarditis 6	Juvenile dermatomyositis 2	Chronic myeloid leukemia 4
Upper respiratory tract infection 5	Crohn’s disease 1	Lymphoma 2
*Salmonella* spp. infection 4	Enthesitis-related arthritis 1	Hepatoblastoma 1
Tropical infection 4	Behçet's disease 1	Ganglioneuroma 1
Meningitis 2	Kikuchi-Fujimoto disease 1	
Enterovirus infection 2	Subcutaneous Sweet syndrome 1	
*Mycoplasma* spp infection 2		
Pyelonephritis 1		
*Clostridium difficile* enteritis 1		
Lemeirre syndrome 1		
Bacterial pericarditis 1		
Adenovirus infection 1		

The clinical characteristics for each etiology of FUO are shown in Table 2. We found that the patients with infections had the shortest duration of fever, whereas the majority of patients with autoimmune diseases and malignancies had a longer duration of fever. Headache and cough were mainly found in infections. Hepatomegaly, splenomegaly, and lymphadenopathy were primarily discovered in malignancies. Arthritis and rash were mostly detected in autoimmune diseases. Other clinical findings, including weight loss, dyspnea, abdominal pain, vomiting, diarrhea, and malaise, were not statistically different among the three etiological categories.

**Table 2:** Baseline characteristics of patients in each group

Parameters	Infection (*n* = 78)	Autoimmune (*n* = 82)	Malignancy (*n* = 80)	*p-*value
Age (years)	5.9 (3.1–12.7)	8.7 (5.2–11.8)	4.8 (3.0–8.9)	0.002*
Female	39 (50)	49 (59.8)	36 (45)	0.16
Duration of fever (days)	12 (8–20.3)	27.5 (15.8–48)	21.5 (14–37.5)	< 0.001*
Clinical manifestations, n (%)				
Headache	13 (16.7)	5 (6.1)	2 (2.5)	0.004*
Cough	47 (60.3)	8 (9.8)	12 (15)	< 0.001*
Poor appetite	35 (44.9)	16 (19.5)	30 (37.5)	0.002*
Vomiting	13 (16.7)	6 (7.3)	7 (8.8)	0.125
Abdominal pain	5 (6.4)	3 (3.7)	8 (10)	0.269
Diarrhea	10 (12.8)	8 (9.8)	3 (3.8)	0.121
Evanescent rash	1 (1.3)	26 (31.7)	0	< 0.001*
Photosensitivity	0	5 (6.1)	0	0.007*
Weight loss	7 (9)	10 (12.2)	17 (21.3)	0.071
Malaise	7 (9)	15 (18.3)	13 (16.3)	0.217
Refuse to walk	0	6 (7.3)	8 (10)	0.021*
Fatigue	4 (5.1)	0	8 (10)	0.014*
Dyspnea	7 (9)	4 (4.9)	1 (1.3)	0.084
Pallor	13 (16.7)	32 (39)	67 (83.8)	< 0.001*
Hepatomegaly	23 (29.5)	35 (42.7)	57 (71.2)	< 0.001*
Splenomegaly	10 (12.8)	15 (18.3)	49 (60)	< 0.001*
Lymphadenopathy	25 (32)	32 (39)	50 (62.5)	< 0.001*
Arthritis	2 (2.5)	50 (60.9)	6 (7.5)	< 0.001*
Pethichiae	2 (2.6)	0	23 (28.8)	< 0.001*
Rash	5 (6.4)	42 (51.2)	2 (2.5)	< 0.001*
Malar rash	0	9 (11)	0	< 0.001*
Discoid rash	0	10 (12.2)	0	< 0.001*
Oral ulcers	1 (1.3)	15 (18.3)	1 (1.3)	< 0.001*
Laboratory data				
Hb (g/dL)	11.1 (9.6–12)	9.7 (8.5–10.8)	7.8 (5.9–9.2)	0.0001*
Hct (%)	33.3 (29.5–36.4)	30.5 (26.7–32.4)	24.1 (18.3–28.4)	0.0001*
WBC (cells/mm^3^)	10,955 (6,650–15,630)	11,780 (6,500–19,300)	10,115 (4,060–37,388)	0.8108
ANC (cells/mm^3^)	7,248 (4,220–10,059)	6,903 (3,802–15,130)	1,280 (468–4,545)	0.0001*
ALC (cells/mm^3^)	2,165 (1,402–3,638)	2,029 (1,421–3,492)	4,381 (1,846–7,575)	0.0001*
Platelet count (cells/mm^3^)	325,500 (228,000–413,000)	403,500 (271,000–595,000)	101,000 (34,500–234,000)	0.0001*

Data presented as median (percentile 25th-percentile 75th). *p-value < 0.05 was set as significance. Hb, hemoglobin; Hct, hematocrit; WBC, white blood cell count; ANC, absolute neutrophil count; ALC, absolute lymphocyte count

We also found that patients with malignancies developed severe anemia and thrombocytopenia more often than patients in the other categories, while leukocyte counts were not statistically different. As more than half of the values for inflammatory markers were missing from the laboratory data, these variables were not analyzed further.

We found some parameters suitable for identifying specific etiologies, including quotidian patterns of fever, evanescent rash and oral ulcers in autoimmune diseases, and the presence of petechiae and progenitors of immature white blood cells in malignancies. These parameters were not normally distributed in all etiological categories, so they were not included in further analyses to avoid bias.

### Parameters associated with etiologies for FUO

Multinomial univariable logistic regression analysis was performed by using infections as a reference. For patients with autoimmune diseases, the duration of fever, headache, and arthritis were associated predictors, whereas cough was found to be an inversely associated parameter. Following multivariable analyses, the predictors of autoimmune diseases were duration of fever 15–30 days (OR = 3.8, 95% CI 1.3–11.1, *p* = 0.015) and > 30 days (OR = 10.3, 95% CI 2.9–35.4, *p* < 0.001), and arthritis (OR = 32.8, 95% CI 6.5–1656.4, *p* < 0.001), as shown in Table 3. Considering malignancies, the duration of fever, particularly that lasting > 30 days, headache, hepatomegaly, splenomegaly, lymphadenopathy, anemia (hemoglobin [Hb] < 7 g/dL), and thrombocytopenia (platelet count < 150,000 cells/mm^3^) were associated factors, whereas cough was an inversely associated parameter similar to those in autoimmune diseases. Following multivariable analysis, the predictors of malignancies were duration of fever > 30 days (OR = 19.4, 95% CI 5.1–73.8, *p* < 0.001), splenomegaly (OR = 5.2, 95% CI 1.8–15.6, *p* = 0.003), lymphadenopathy (OR = 4.2, 95% CI 1.6–11.2, *p* = 0.004), anemia (OR = 9.2, 95% CI 2.3–36.9, *p* = 0.002) and thrombocytopenia (OR = 10, 95% CI 3.3–30.1, *p* < 0.001), as shown in Table 4. The presence of cough reduced the likelihood of etiologies of autoimmune diseases (OR = 0.1, 95% CI 0.1–0.4, *p* < 0.001) and malignancies (OR = 0.1, 95% CI 0.04–0.4, *p* < 0.001). The Hausman–McFadden test for the IIA in the autoimmune diseases category yielded a χ^2^ value of 8.36 with a *p*-value of 0.493, suggesting no significant evidence against the IIA assumption. Similarly, in the malignancies category, the test statistic was χ^2^ = 5.33 with a *p*-value of 0.8044, indicating no significant violation of the IIA assumption. These results suggest that the multinomial logistic regression model satisfies the IIA assumption for autoimmune diseases and malignancies.

**Table 3:** Predictors of autoimmune diseases in patients with fever of unknown origin

Clinical parameters	Univariable		Multivariable	
	OR (95% CI)	*p-*value	OR (95% CI)	*p-*value
Duration of fever				
15–30 days	4.4 (1.9–9.8)	< 0.001*	3.8 (1.3–11.1)	0.015*
> 30 days	15.9 (6.1–41.4)	< 0.001*	10.3 (2.9–35.4)	< 0.001*
Having cough	0.1 (0.03–0.2)	< 0.001*	0.1 (0.1–0.4)	< 0.001*
Headache	0.4 (0.1–0.9)	0.042*		
Hepatomegaly	1.8 (0.9–3.4)	0.1		
Splenomegaly	1.5 (0.6–3.6)	0.3	2.3 (0.7–7.4)	0.148
Lymphadenopathy	1.4 (0.7–2.6)	0.358	1.4 (0.6–3.7)	0.463
Arthritis	59.4 (13.6–258.9)	< 0.001*	32.8 (6.5–166.4)	< 0.001*
Anemia^†^	1.5 (0.4–5.4)	0.569	2.3 (0.5–10.8)	0.298
Thrombocytopenia^‡^	0.6 (0.2–1.6)	0.27	0.9 (0.3–3.2)	0.878

^*^ *p*-value < 0.05 was set as significance; ^†^Hemoglobin (Hb) < 7 g/dL, ^‡^Platelets < 150,000 cells/mm^3^

**Table 4:** Predictors of malignancy in patients with fever of unknown origin

Clinical parameters	Univariable		Multivariable	
	OR (95% CI)	*p-*value	OR (95% CI)	*p-*value
Duration of fever				
15–30 days	2.2 (1–4.8)	0.04*	3.0 (0.9–9.4)	0.055
> 30 days	8.4 (3.4–21)	< 0.001*	19.4 (5.1–73.8)	< 0.001*
Having cough	0.1 (0.1–0.3)	< 0.001*	0.1 (0.04–0.4)	< 0.001*
Headache	0.1 (0.3–0.6)	0.008*		
Hepatomegaly	5.9 (3–11.8)	< 0.001*		
Splenomegaly	10.2 (4.6–22.7)	< 0.001*	5.2 (1.8–15.6)	0.003*
Lymphadenopathy	3.5 (1.8–6.8)	< 0.001*	4.2 (1.6–11.2)	0.004*
Arthritis	3.1 (0.6–15.8)	0.2	2.9 (0.4–19.5)	0.277
Anemia^†^	12.9 (4.3–39)	< 0.001*	9.2 (2.3–36.9)	0.002*
Thrombocytopenia^‡^	10.7 (4.9–23.5)	< 0.001*	10.0 (3.3–30.1)	< 0.001*

^*^ p-value < 0.05 was set as significance; ^†^Hemoglobin (Hb) < 7 g/dL, ^‡^Platelets < 150,000 cells/mm^3^

### Developing the prediction model

Seven associated parameters in both autoimmune diseases and malignancies were included in the prediction model. The prediction model for autoimmune diseases compared with infections used the following equation: − 1.5 + (3.5 × arthritis) + (1.3 × duration of fever 15–30 days) + (2.3 × duration of fever > 30 days) + (− 1.9 × cough) + (0.8 × splenomegaly) + (0.4 × lymphadenopathy) + (0.8 × Hb < 7 g/dL) + (− 0.1 × platelet count < 150,000 cells/mm^3^). The prediction model for malignancies compared with infections used the following equation: − 2.8 + (1.1 × arthritis) + (1.1 × duration of fever 15–30 days) + (3 × duration of fever > 30 days) + (− 2.1 × cough) + (1.7 × splenomegaly) + (1.4 × lymphadenopathy) + (2.2 × Hb < 7 g/dL) + (2.3 × platelet count < 150,000 cells/mm^3^). A computerized prediction model was then constructed using predictive margin analysis (Supplemental Fig. 1). The prediction model provides the probability for each disease category, allowing physicians to use their clinical judgment to assess the possible cause of FUO and to determine the need for further investigation.

### Performance of the prediction model

**Fig. 1:**
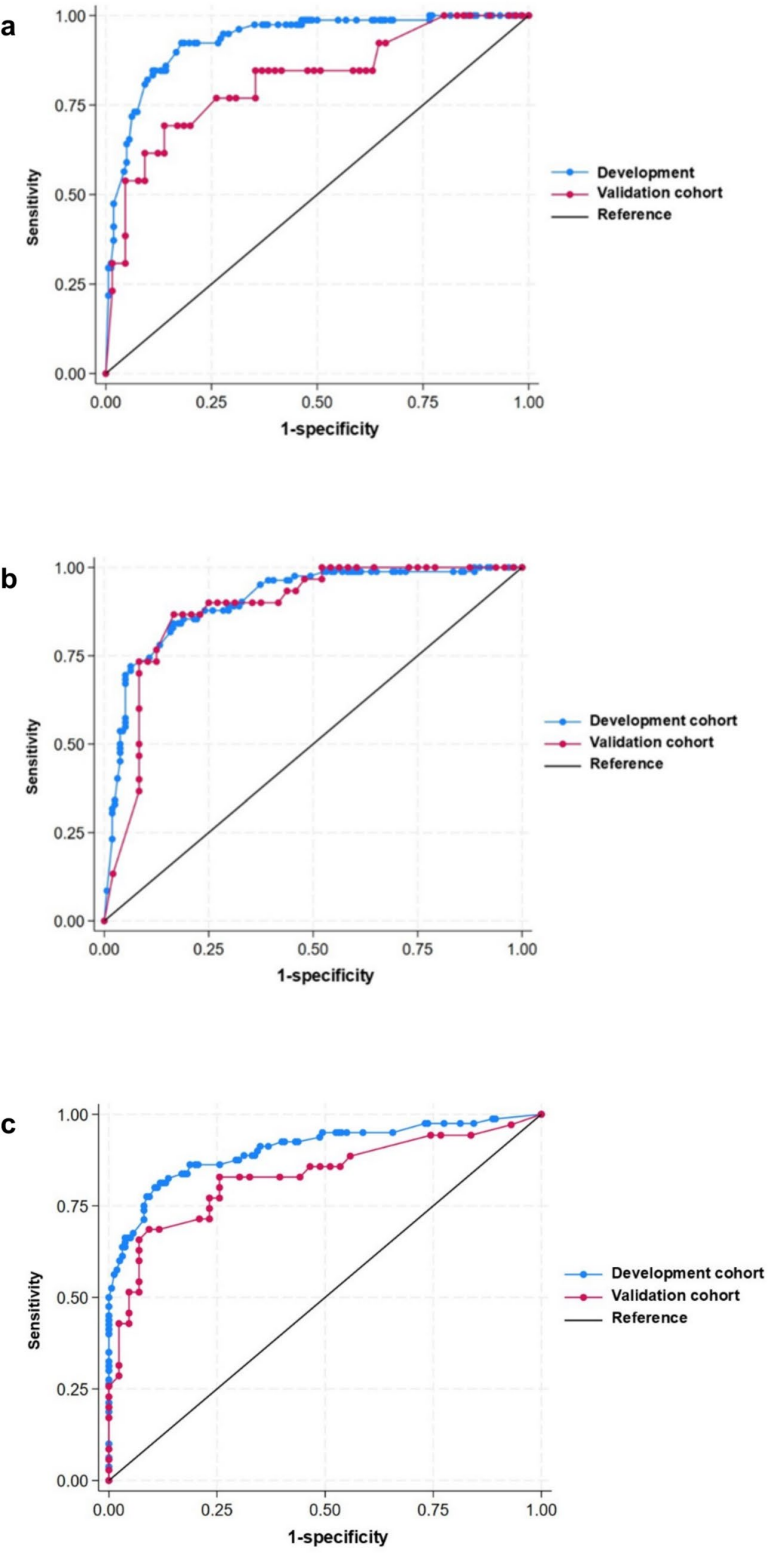
Receiver operating characteristic curves of the development cohort (*n* = 240) and validation cohort (*n* = 78), in which (**a**) represents infections, (**b**) represents autoimmune diseases, and (**c**) represents malignancies

We evaluated the prediction model using the AUC (Fig. 1). In the development cohort, the AUC was 0.91 (95% CI 0.90–0.96) for infections, 0.90 (95% CI 0.86–0.94) for autoimmune diseases, and 0.90 (95% CI 0.86–0.95) for malignancies. In the validation cohort, the AUC was 0.82 (95% CI 0.68–0.96) for infections, 0.88 (95% CI 0.80–0.96) for autoimmune diseases, and 0.83 (95% CI 0.73–0.93) for malignancies.

## Discussion

This study is the first to develop a prediction model for approaching the etiologies of FUO in children by integrating clinical parameters from medical histories, physical examinations, and initial laboratory results. The prediction model was based on seven key parameters: duration of fever, cough, arthritis, splenomegaly, lymphadenopathy, anemia, and thrombocytopenia. Following multinomial logistic regression and predictive margin analyses, the model demonstrated good performance, particularly when distinguishing autoimmune diseases and malignancies, as assessed by the AUC. Previous studies described clinical parameters and FUO etiologies but did not demonstrate significant differences among these parameters, likely due to differences in study design and variable selection [1, 4, 7]. They focused on demographic factors or laboratory findings obtained later in the diagnostic process. In contrast, our study emphasized early clinical symptoms, physical examination findings, and basic laboratory tests, which are routinely available at initial presentation. This approach aligns with our aim of developing an early prediction model to support clinical decision-making before initiating extensive diagnostic workups. This model may help reduce unnecessary investigations and inappropriate treatments, especially in resource-limited settings. Our findings emphasize the potential utility of combining these early clinical markers into a systematic tool to aid diagnosis and reduce diagnostic uncertainty.

We found notable similarities and differences when comparing our results to previous studies. Consistent with the findings of Hu et al., the shortest duration of fever was observed in the infections category [7]. Cough emerged as a significant predictor in our model, likely because respiratory tract infections constituted the top three diagnoses in the infections category. Unlike prior studies, which frequently identified leukocytosis in autoimmune conditions due to various diseases, such as Kawasaki disease and inflammatory bowel disease [1, 12], our study did not observe this result. This discrepancy is likely attributable to the predominance of SJIA and SLE in our autoimmune diseases category, with SLE often presenting with leukopenia instead of leukocytosis [4, 12]. Additionally, prolonged fever was consistently associated with autoimmune conditions across various studies, aligning with our results [4, 7]. For malignancy-related FUO, our findings, characterized by prolonged fever, severe anemia, and thrombocytopenia, are consistent with previous research, likely due to the high prevalence of acute lymphoblastic leukemia involving bone marrow suppression and cytopenia [7, 12].

The overall performance of this prediction model was good, particularly when identifying autoimmune diseases, followed by malignancies. However, a difference in AUC for infections was observed between the validation and development cohorts. This discrepancy may be attributed to the relatively small number of patients with infections in the validation cohort and slight differences in baseline characteristics between the two cohorts. Nevertheless, this prediction model is particularly beneficial for patients with autoimmune diseases and malignancies, as these conditions can be life-threatening, especially in those presenting with prolonged fever. When differentiating these two conditions is challenging, physicians must decide on the appropriate course for further investigation. In such cases, a simultaneous workup for both conditions may be necessary to prevent delays in diagnosis, which could otherwise lead to increased morbidity and mortality.

This prediction model was developed using historical data, physical examination findings, and basic laboratory investigations, such as CBC. Other laboratory markers, such as uric acid and lactate dehydrogenase for malignancies or procalcitonin and blood cultures for infections, were not included due to missing data, as these tests were performed only for specific etiologies. As a result, this prediction model does not provide definitive diagnoses but serves as a clinical tool to support informed decision-making, which will guide physicians in determining the need for further investigations to identify specific etiologies.

The limitations of this study should be noted. First, this was a single-center study conducted in a tertiary care hospital, which may limit generalizability to the broader population of children in other regions. The etiologies of FUO in this study reflect both local epidemiology and our role as a tertiary referral center. For example, tuberculosis is endemic in Thailand; Epstein-Barr virus is frequently detected during thorough FUO workups, and lower respiratory tract infections are common in children. Similarly, SJIA and SLE are among the most prevalent autoimmune diseases in Asian pediatric populations. Acute lymphoblastic leukemia is the most common childhood malignancy and is frequently referred to our institution, whereas solid tumors were underrepresented, likely due to their lower likelihood of presenting with FUO alone. Consequently, the model may be less sensitive for detecting solid malignancies. Second, a retrospective study design in nature caused missing data, particularly inflammatory markers such as C-reactive protein and erythrocyte sedimentation rate, which prevented the inclusion of potentially essential variables in the prediction model. Third, patients without a confirmed final diagnosis were excluded from the model development. Since our primary aim was to construct a classification model that categorizes pediatric FUO cases into three specific etiologic groups, including infections, autoimmune diseases, and malignancies, patients with an unresolved etiology would introduce classification uncertainty and compromise model validity. In our cohort, only two patients remained undiagnosed, both of whom experienced spontaneous resolution of fever, likely due to an unspecified viral illness. These cases were not included in the infectious group due to a lack of confirmed etiology. The low number of undiagnosed cases may reflect the comprehensive diagnostic capabilities available at our tertiary care facility. However, we recognize that in routine clinical settings, a proportion of FUO cases remain undiagnosed. Therefore, future multi-center studies with a prospective study design, including undiagnosed patients, are needed to perform external validation and refine this prediction model.

Despite these limitations, this study’s findings have significant implications for clinical practice. The prediction model developed here can become a complementary tool to guide clinicians toward informed, data-driven decision-making, which will facilitate earlier diagnoses and reduce unnecessary testing and treatments. However, this model is not intended to replace clinical judgment or the diagnostic reasoning of physicians. Instead, it serves as a supportive tool by estimating the probability of various diagnostic possibilities based on clinical and laboratory data. The final diagnosis and management should always be grounded in comprehensive clinical evaluation and physician expertise.

In conclusion, key clinical parameters, including duration of fever, cough, splenomegaly, arthritis, lymphadenopathy, hemoglobin, and platelet levels, were identified as significant predictors of the etiologies of FUO in children. These factors were integrated into a prediction model designed to guide clinicians in differentiating the underlying causes of pediatric FUO, encompassing infections, autoimmune diseases, and malignancies.

The prediction model has demonstrated promise as a practical clinical tool by aiding early diagnoses and supporting evidence-based decision-making in managing pediatric FUO. This model might reduce diagnostic delays, minimize unnecessary testing, and improve patient outcomes by providing a systematic approach to interpreting clinical and basic laboratory data. Future multi-center studies are warranted to validate and enhance the applicability of the model across diverse pediatric populations.

## Supplementary Information

Below is the link to the electronic supplementary material.

Supplementary file1 (DOCX 278 KB)

## Data Availability

The data generated or analyzed during this study are included in this article and its supplementary information files.

## Abbreviations

FUO: Fever of unknown origin
ICD-10: International Classification of Diseases, Tenth Revision
AUC: Area under the Receiver-Operating Characteristic curve
SJIA: Systemic juvenile idiopathic arthritis
SLE: Systemic lupus erythematosus
Hb: Hemoglobin
