# Supplementary material for: Prediction model for etiology of fever of unknown origin in children
Source: Eur J Pediatr. 2025 Jun 19;184(7):429. doi: 10.1007/s00431-025-06277-4 (PMC12176973; doi:10.1007/s00431-025-06277-4)
Supplement: Supplementary file 1 — Supplementary file1 (DOCX 278 KB) [file 431_2025_6277_MOESM1_ESM.docx]

**Prediction model for etiology of fever of unknown origin in children**

Pannachet Rienvichit, Butsabong Lerkvaleekul, Nopporn Apiwattanakul, Samart Pakakasama, Sasivimol Rattanasiri, Soamarat Vilaiyuk*

*Corresponding author: soamarat21@hotmail.com

**Supplemental Fig. 1** The computerized prediction model was constructed by predictive margin analysis. The table below illustrates how the prediction model functions. After entering patient data into this model (a), the predicted probabilities for each diagnostic group are displayed in the output table (b).


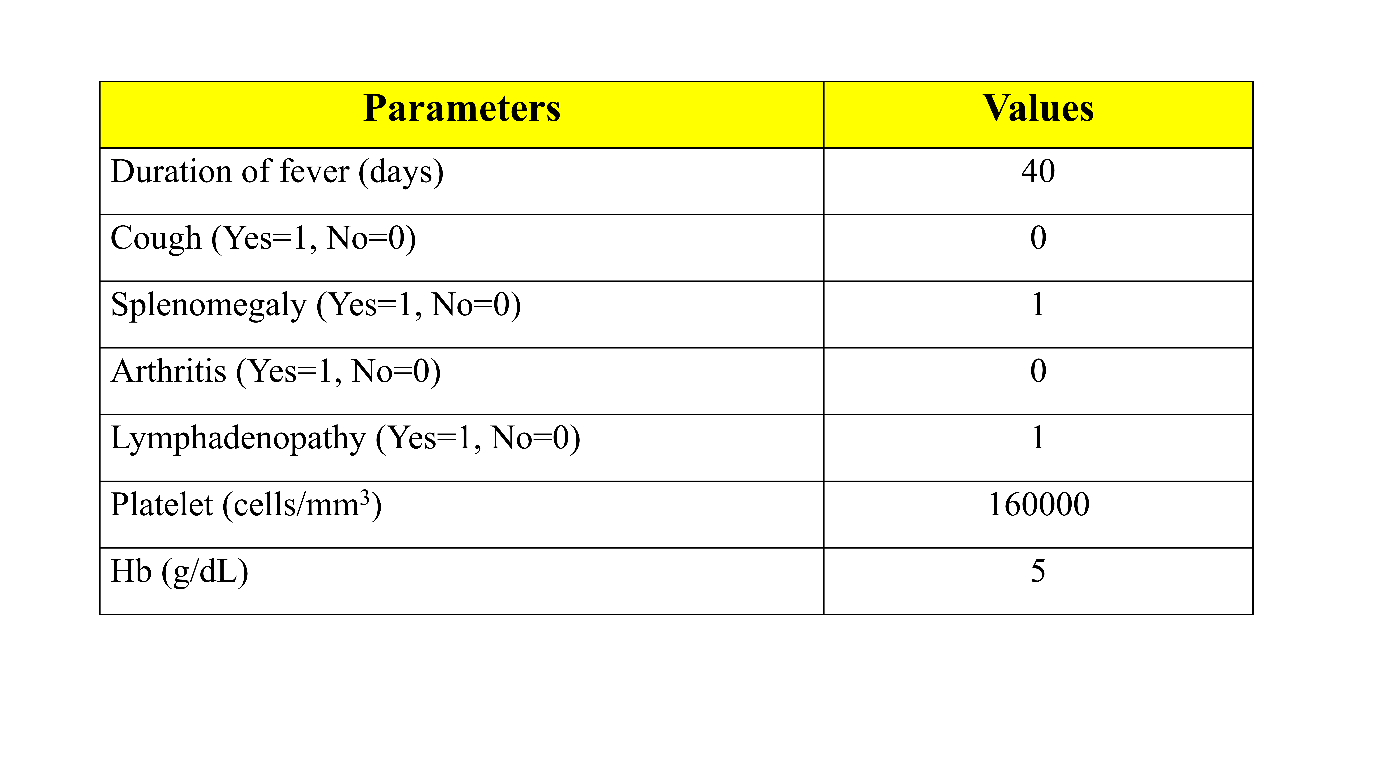


**a.**


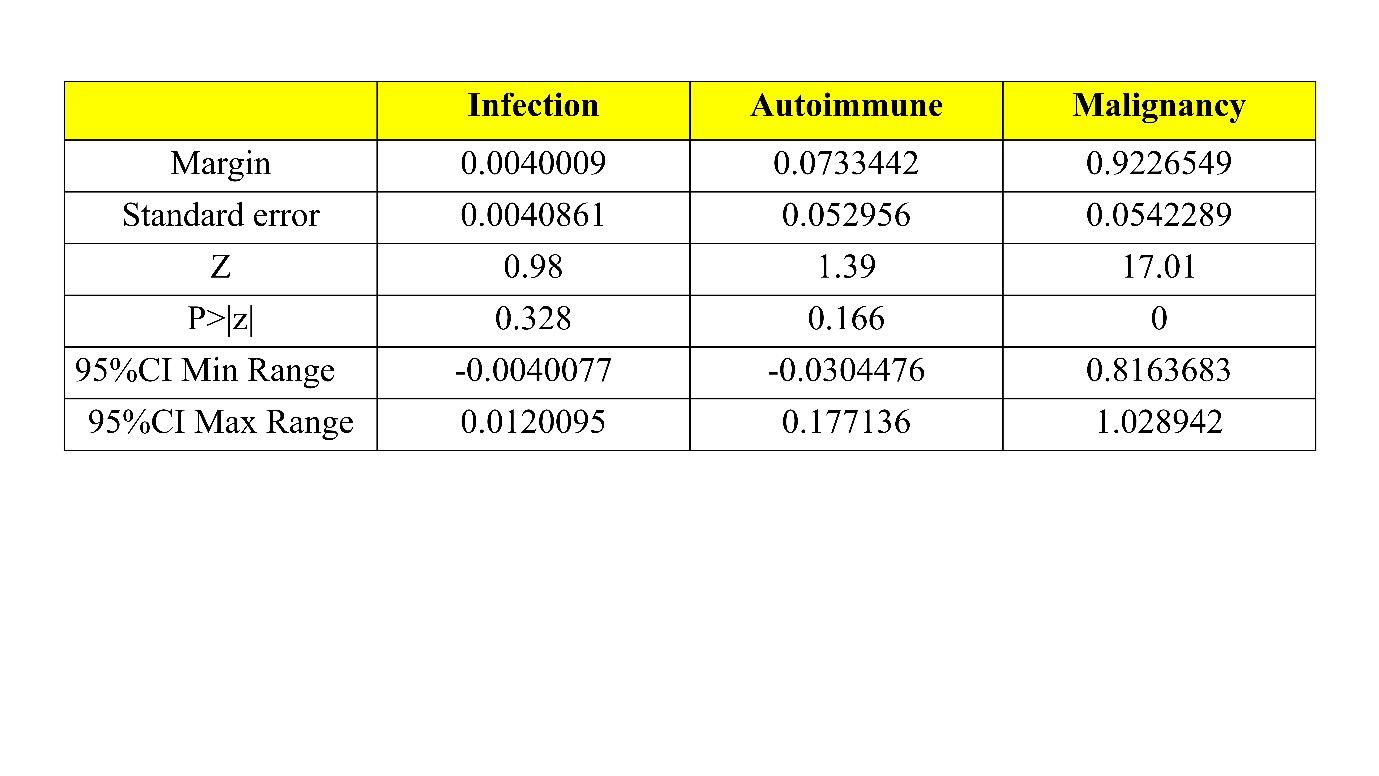


**b.**

The “Margin” values x 100 = probability

From this data, this patient has probabilities to be infection = 0.4%, autoimmune = 7.3%, and malignancy = 92.3%

**Supplemental Table 1** ICD-10 Codes

| ICD-10 Code | Description |
| --- | --- |
| A020 | Salmonella enteritis |
| A021 | Salmonella septicaemia |
| A022 | Localized salmonella infections |
| A028 | Other specified salmonella infections |
| A029 | Salmonella infection, unspecified |
| A15 | Respiratory tuberculosis, bacteriologically and histologically confirmed |
| A150 | Tuberculosis of lung, confirmed by sputum microscopy with or without culture |
| A151 | Tuberculosis of lung, confirmed by culture only |
| A152 | Tuberculosis of lung, confirmed histologically |
| A153 | Tuberculosis of lung, confirmed by unspecified means |
| A154 | Tuberculosis of intrathoracic lymph nodes, confirmed bacteriologically and histologically confirmed |
| A155 | Tuberculosis of larynx, trachea and bronchus, confirmed bacteriologically and histologically confirmed |
| A156 | Tuberculous pleurisy, confirmed bacteriologically and histologically |
| A158 | Other respiratory tuberculosis, confirmed bacteriologically and histologically |
| A159 | Respiratory tuberculosis unspecified, confirmed bacteriologically and histologically |
| A160 | Tuberculosis of lung, bacteriologically and histologically negative |
| A161 | Tuberculosis of lung, bacteriological and histological examination not done |
| A162 | Tuberculosis of lung, without mention of bacteriological or histological confirmation |
| A163 | Tuberculosis of intrathoracic lymph nodes, without mention of bacteriological or histological confirmation |
| A164 | Tuberculosis of larynx, trachea and bronchus, without mention of bacteriological or histological confirmation |
| A165 | Tuberculous pleurisy, without mention of bacteriological or histological confirmation |
| A167 | Primary respiratory tuberculosis without mention of bacteriological or or histological confirmation |
| A168 | Other respiratory tuberculosis, without mention of bacteriological or histological confirmation |
| A169 | Respiratory tuberculosis unspecified, without mention of bacteriological or histological confirmation |
| A170 | Tuberculous meningitis (G01*) |
| A171 | Meningeal tuberculoma (G07*) |
| A178 | Other tuberculosis of nervous system |
| A179 | Tuberculosis of nervous system, unspecified (G99.8*) |
| A180 | Tuberculosis of bones and joints |
| A181 | Tuberculosis of genitourinary system |
| A182 | Tuberculous peripheral lymphadenopathy |
| A183 | Tuberculosis of intestines, peritoneum and mesenteric glands |
| A184 | Tuberculosis of skin and subcutaneous tissue |
| A185 | Tuberculosis of eye |
| A186 | Tuberculosis of ear |
| A188 | Tuberculosis of other specified organs |
| A190 | Acute miliary tuberculosis of a single specified site |
| A191 | Acute miliary tuberculosis of multiple sites |
| A192 | Acute miliary tuberculosis, unspecified |
| A198 | Other miliary tuberculosis |
| A199 | Miliary tuberculosis, unspecified |
| A28 | Other zoonotic bacterial diseases, not elsewhere classified |
| A281 | Cat-scratch disease |
| A38 | Scarlet fever |
| A490 | Staphylococcal infection, unspecified |
| A491 | Streptococcal infection, unspecified |
| A492 | Haemophilus influenzae infection, unspecified |
| A493 | Mycoplasma infection, unspecified |
| A498 | Other bacterial infections of unspecified site |
| A499 | Bacterial infection, unspecified |
| A68 | Relapsing fevers |
| A681 | Tick-borne relapsing fever |
| A75 | Typhus fever |
| A750 | Epidemic louse-borne typhus fever due to Rickettsia prowazekii |
| A751 | Recrudescent typhus [Brill's disease] |
| A752 | Typhus fever due to Rickettsia typhi |
| A753 | Typhus fever due to Rickettsia tsutsugamushi |
| A759 | Typhus fever, unspecified |
| A84 | Tick-borne viral encephalitis |
| A840 | Far Eastern tick-borne encephalitis [Russian spring-summer encephalitis] |
| A841 | Central European tick-borne encephalitis |
| A848 | Other tick-borne viral encephalitis |
| A849 | Tick-borne viral encephalitis, unspecified |
| A850 | Enteroviral encephalitis (G05.1*) |
| A858 | Other specified viral encephalitis |
| A878 | Other viral meningitis |
| A879 | Viral meningitis, unspecified |
| A880 | Enteroviral exanthematous fever [Boston exanthem] |
| A881 | Epidemic vertigo |
| A888 | Other specified viral infections of the central nervous system |
| A97 | Dengue |
| A970 | Dengue without warning signs |
| A971 | Dengue with warning signs |
| A972 | Severe Dengue |
| A979 | Dengue, unspecified |
| B081 | Molluscum contagiosum |
| B082 | Exanthema subitum [sixth disease] |
| B083 | Erythema infectiosum [fifth disease] |
| B084 | Enteroviral vesicular stomatitis with exanthem |
| B085 | Enteroviral vesicular pharyngitis |
| B088 | Other specified viral infections characterized by skin and mucous membrane lesions |
| B171 | Acute hepatitis C |
| B172 | Acute hepatitis E |
| B178 | Other specified acute viral hepatitis |
| B179 | Acute viral hepatitis, unspecified |
| B181 | Chronic viral hepatitis B without delta-agent |
| B182 | Chronic viral hepatitis C |
| B189 | Chronic viral hepatitis, unspecified |
| B199 | Unspecified viral hepatitis without coma |
| B250 | Cytomegaloviral pneumonitis (J17.1*) |
| B251 | Cytomegaloviral hepatitis (K77.0*) |
| B258 | Other cytomegaloviral diseases |
| B259 | Cytomegaloviral disease, unspecified |
| B270 | Gammaherpesviral mononucleosis |
| B271 | Cytomegaloviral mononucleosis |
| B278 | Other infectious mononucleosis |
| B279 | Infectious mononucleosis, unspecified |
| B340 | Adenovirus infection, unspecified |
| B341 | Enterovirus infection, unspecified |
| B342 | Coronavirus infection, unspecified |
| B343 | Parvovirus infection, unspecified |
| B348 | Other viral infections of unspecified site |
| B349 | Viral infection, unspecified |
| B519 | Plasmodium vivax malaria without complication |
| B529 | Plasmodium malariae malaria without complication |
| B960 | Mycoplasma pneumoniae [M. pneumoniae] as the cause of diseases classified to other chapters |
| B961 | Klebsiella pneumoniae [K.pneumoniae] as the cause of diseases classified to other chapters |
| B962 | Escherichia coli [E. coli] as the cause of diseases classified to other chapters |
| B963 | Haemophilus influenzae [H. influenzae] as the cause of diseases classified to other chapters |
| B964 | Proteus (mirabilis)(morganii) as the cause of diseases classified to other chapters |
| B965 | Pseudomonas (aeruginosa)(mallei)(pseudomallei) as the cause of diseases classified to other chapters |
| B966 | Bacillus fragilis [B. fragilis] as the cause of diseases classified to other chapters |
| B968 | Other specified bacterial agents as the cause of diseases classified to other chapters |
| B970 | Adenovirus as the cause of diseases classified to other chapters |
| B971 | Enterovirus as the cause of diseases classified to other chapters |
| B972 | Coronavirus as the cause of diseases classified to other chapters |
| B973 | Retrovirus as the cause of diseases classified to other chapters |
| B974 | Respiratory syncytial virus as the cause of diseases classified to other chapters |
| B976 | Parvovirus as the cause of diseases classified to other chapters |
| B977 | Papillomavirus as the cause of diseases classified to other chapters |
| B978 | Other viral agents as the cause of diseases classified to other chapters |
| C810 | Nodular lymphocyte predominant Hodgkin lymphoma |
| C811 | Nodular sclerosis classical Hodgkin lymphoma |
| C812 | Mixed cellularity classical Hodgkin lymphoma |
| C813 | Lymphocytic depletion classical Hodgkin lymphoma |
| C814 | Lymphocyte-rich classical Hodgkin lymphoma |
| C817 | Other classical Hodgkin lymphoma |
| C819 | Hodgkin lymphoma, unspecified |
| C822 | Follicular lymphoma grade III, unspecified |
| C827 | Other types of follicular lymphoma |
| C829 | Follicular lymphoma, unspecified |
| C830 | Small cell B-cell lymphoma |
| C831 | Mantle cell lymphoma |
| C832 | Mixed small and large cell (diffuse) non-Hodgkin's lymphoma |
| C833 | Diffuse large B-cell lymphoma |
| C835 | Lymphoblastic (diffuse) |
| C836 | Undifferentiated (diffuse) non-Hodgkin's lymphoma |
| C837 | Burkitt's lymphoma |
| C838 | Other non-follicular lymphoma |
| C839 | Non-follicular diffuse non-Hodgkin lymphoma, unspecified |
| C840 | Mycosis fungoides |
| C842 | Peripheral and cutaneous T-cell lymphomas ,T-zone lymphoma |
| C844 | Peripheral T-cell lymphoma, not classified |
| C845 | Other and unspecified T-cell lymphomas |
| C846 | Anaplastic large cell lymphoma, ALK-positive |
| C847 | Anaplastic large cell lymphoma, ALK-negative |
| C848 | Cutaneous T-cell lymphoma, unspecified |
| C849 | Mature T/NK-cell lymphoma, unspecified |
| C851 | B-cell lymphoma, unspecified |
| C857 | Other specified types of non-Hodgkin's lymphoma |
| C859 | Non-Hodgkin's lymphoma, unspecified type |
| C860 | Extranodal NK/T-cell lymphoma, nasal type |
| C863 | Subcutaneous panniculitis-like T-cell lymphoma |
| C864 | Blastic NK-cell lymphoma |
| C866 | Primary cutaneous CD30-positive T-cell proliferations |
| C910 | Acute lymphoblastic leukaemia |
| C911 | Chronic lymphocytic leukaemia of B-cell type |
| C915 | Adult T-cell ymphoma/leukaemia (HTLV-1-associated) |
| C918 | Mature B-cell leukaemia Burkitt-type |
| C919 | Lymphoid leukaemia, unspecified |
| C920 | Acute myeloblastic leukaemia [AML] |
| C921 | Chronic myeloid leukaemia |
| C923 | Myeloid sarcoma |
| C924 | Acute promyelocytic leukaemia [PML] |
| C925 | Acute myelomonocytic leukaemia |
| C929 | Myeloid leukaemia, unspecified |
| C950 | Acute leukaemia of unspecified cell type |
| C951 | Chronic leukaemia of unspecified cell type |
| C957 | Other leukaemia of unspecified cell type |
| C959 | Leukaemia, unspecified |
| C960 | Multifocal and multisystemic (disseminated) Langerhanscell histiocytosis [Letterer-Siwe disease] |
| C961 | Malignant histiocytosis |
| C962 | Malignant mast cell tumour |
| C963 | True histiocytic lymphoma |
| C965 | Multifocal and unisystemic Langerhans-cell histiocytosis |
| C966 | Unifocal Langerhans-cell histiocytosis |
| C968 | Histiocytic sarcoma |
| C969 | Malignant neoplasm of lymphoid, haematopoietic and related tissue, unspecified |
| D61 | Other aplastic anaemias |
| D610 | Constitutional aplastic anaemia |
| D611 | Drug-induced aplastic anaemia |
| D612 | Aplastic anaemia due to other external agents |
| D613 | Idiopathic aplastic anaemia |
| D618 | Other specified aplastic anaemias |
| D619 | Aplastic anaemia, unspecified |
| D73 | Diseases of spleen |
| D733 | Abscess of spleen |
| D760 | Langerhans' cell histiocytosis, not elsewhere classified |
| D761 | Haemophagocytic lymphohistiocytosis |
| D762 | Haemophagocytic syndrome, infection-associated |
| D763 | Other histiocytosis syndromes |
| D868 | Sarcoidosis of other and combined sites |
| D869 | Sarcoidosis, unspecified |
| G06 | Intracranial and intraspinal abscess and granuloma |
| G060 | Intracranial abscess and granuloma |
| H700 | Acute mastoiditis |
| H701 | Chronic mastoiditis |
| H709 | Mastoiditis, unspecified |
| H750 | Mastoiditis in infectious and parasitic diseases classified elsewhere |
| I00 | Rheumatic fever without mention of heart involvement |
| I01 | Rheumatic fever with heart involvement |
| I010 | Acute rheumatic pericarditis |
| I011 | Acute rheumatic endocarditis |
| I012 | Acute rheumatic myocarditis |
| I018 | Other acute rheumatic heart disease |
| I019 | Acute rheumatic heart disease, unspecified |
| I09 | Other rheumatic heart diseases |
| I091 | Rheumatic diseases of endocardium, valve unspecified |
| I33 | Acute and subacute endocarditis |
| I330 | Acute and subacute infective endocarditis |
| I339 | Acute endocarditis, unspecified |
| I38 | Endocarditis, valve unspecified |
| I39 | Endocarditis and heart valve disorders in diseases classified elsewhere |
| I390 | Mitral valve disorders in diseases classified elsewhere |
| I391 | Aortic valve disorders in diseases classified elsewhere |
| I392 | Tricuspid valve disorders in diseases classified elsewhere |
| I393 | Pulmonary valve disorders in diseases classified elsewhere |
| I394 | Multiple valve disorders in diseases classified elsewhere |
| I398 | Endocarditis, valve unspecified, in diseases classified elsewhere |
| J010 | Acute maxillary sinusitis |
| J011 | Acute frontal sinusitis |
| J012 | Acute ethmoidal sinusitis |
| J014 | Acute pansinusitis |
| J018 | Other acute sinusitis |
| J019 | Acute sinusitis, unspecified |
| J150 | Pneumonia due to Klebsiella pneumoniae |
| J151 | Pneumonia due to Pseudomonas |
| J152 | Pneumonia due to Staphylococcus |
| J154 | Pneumonia due to other streptococci |
| J155 | Pneumonia due to Escherichia coli |
| J156 | Pneumonia due to other aerobic gram-negative bacteria |
| J157 | Pneumonia due to Mycoplasma pneumoniae |
| J158 | Other bacterial pneumonia |
| J159 | Bacterial pneumonia, unspecified |
| J20 | Acute bronchitis |
| J200 | Acute bronchitis due to Mycoplasma pneumoniae |
| J201 | Acute bronchitis due to Haemophilus influenzae |
| J202 | Acute bronchitis due to streptococcus |
| J203 | Acute bronchitis due to coxsackie virus |
| J204 | Acute bronchitis due to parainfluenza virus |
| J205 | Acute bronchitis due to respiratory syncytial virus |
| J206 | Acute bronchitis due to rhinovirus |
| J207 | Acute bronchitis due to echovirus |
| J208 | Acute bronchitis due to other specified organisms |
| J209 | Acute bronchitis, unspecified |
| J320 | Chronic maxillary sinusitis |
| J321 | Chronic frontal sinusitis |
| J322 | Chronic ethmoidal sinusitis |
| J323 | Chronic sphenoidal sinusitis |
| J324 | Chronic pansinusitis |
| J328 | Other chronic sinusitis |
| J329 | Chronic sinusitis, unspecified |
| K50 | Crohn's disease [regional enteritis] |
| K500 | Crohn's disease of small intestine |
| K501 | Crohn's disease of large intestine |
| K508 | Other Crohn's disease |
| K509 | Crohn's disease, unspecified |
| K510 | Ulcerative (chronic) enterocolitis |
| K511 | Ulcerative (chronic) ileocolitis |
| K512 | Ulcerative (chronic) proctitis |
| K513 | Ulcerative (chronic) rectosigmoiditis |
| K515 | Mucosal proctocolitis |
| K518 | Other ulcerative colitis |
| K519 | Ulcerative colitis, unspecified |
| K75 | Other inflammatory liver diseases |
| K750 | Abscess of liver |
| L51 | Erythema multiforme |
| L511 | Bullous erythema multiforme |
| L920 | Granuloma annulare |
| L922 | Granuloma faciale [eosinophilic granuloma of skin] |
| L923 | Foreign body granuloma of the skin and subcutaneous tissue |
| L928 | Other granulomatous disorders of skin and subcutaneous tissue |
| L929 | Granulomatous disorder of skin and subcutaneous tissue, unspecified |
| M080 | Juvenile rheumatoid arthritis |
| M0800 | Juvenile rheumatoid arthritis : multiple sites |
| M0801 | Juvenile rheumatoid arthritis Shoulder region, clavicle, scapula, acromioclavicular, glenohumeral, sternoclavicular, (joints) |
| M0802 | Juvenile rheumatoid arthritis Upper arm, humerus, elbow joint |
| M0803 | Juvenile rheumatoid arthritis Forearm, radius, ulna, wrist joint |
| M0804 | Juvenile rheumatoid arthritis Hand, carpus, fingers, metacarpus, joints between these bones |
| M0805 | Juvenile rheumatoid arthritis Pelvic region and thigh, buttock, femur, pelvis, hip (joint), sacroiliac joint |
| M0806 | Juvenile rheumatoid arthritis Lower leg, fibula, tibia, knee joint |
| M0807 | Juvenile rheumatoid arthritis Ankle and foot, metatarsus, tarsus, toes, ankle joint, other joints in foot |
| M0808 | Juvenile rheumatoid arthritis Other, head, neck, ribs, skull, trunk, vertebral column |
| M0809 | Juvenile rheumatoid arthritis : site unspecified |
| M081 | Juvenile ankylosing spondylitis |
| M0810 | Juvenile ankylosing spondylitis, multiple sites |
| M0812 | Juvenile ankylosing spondylitis Upper arm, humerus, elbow joint |
| M0813 | Juvenile ankylosing spondylitis Forearm, radius, ulna, wrist joint |
| M0814 | Juvenile ankylosing spondylitis Hand, carpus, fingers, metacarpus, joints between these bones |
| M0815 | Juvenile ankylosing spondylitis Pelvic region and thigh, buttock, femur, pelvis, hip (joint), sacroiliac joint |
| M0816 | Juvenile ankylosing spondylitis Lower leg, fibula, tibia, knee joint |
| M0817 | Juvenile ankylosing spondylitis Ankle and foot, metatarsus, tarsus, toes, ankle joint, other joints in foot |
| M0819 | Juvenile ankylosing spondylitis, site unspecified |
| M082 | Juvenile arthritis with systemic onset [Still] |
| M0820 | Juvenile arthritis with systemic onset [Still], multiple sites |
| M0821 | Juvenile arthritis with systemic onset Shoulder region, clavicle, scapula, acromioclavicular, glenohumeral, sternoclavicular, (joints) |
| M0822 | Juvenile arthritis with systemic onset Upper arm, humerus, elbow joint |
| M0823 | Juvenile arthritis with systemic onset Forearm, radius, ulna, wrist joint |
| M0824 | Juvenile arthritis with systemic onset Hand, carpus, fingers, metacarpus, joints between these bones |
| M0825 | Juvenile arthritis with systemic onset Pelvic region and thigh, buttock, femur, pelvis, hip (joint), sacroiliac joint |
| M0826 | Juvenile arthritis with systemic onset Lower leg, fibula, tibia, knee joint |
| M0827 | Juvenile arthritis with systemic onset Ankle and foot, metatarsus, tarsus, toes, ankle joint, other joints in foot |
| M0828 | Juvenile arthritis with systemic onset Other, head, neck, ribs, skull, trunk, vertebral column |
| M0829 | Juvenile arthritis with systemic onset [Still], site unspecified |
| M083 | Juvenile polyarthritis (seronegative) |
| M0830 | Juvenile polyarthritis (seronegative), multiple sites |
| M0831 | Juvenile polyarthritis (seronegative) Shoulder region, clavicle, scapula, acromioclavicular, glenohumeral, sternoclavicular, (joints) |
| M0832 | Juvenile polyarthritis (seronegative) Upper arm, humerus, elbow joint |
| M0833 | Juvenile polyarthritis (seronegative) Forearm, radius, ulna, wrist joint |
| M0834 | Juvenile polyarthritis (seronegative) Hand, carpus, fingers, metacarpus, joints between these bones |
| M0835 | Juvenile polyarthritis (seronegative) Pelvic region and thigh, buttock, femur, pelvis, hip (joint), sacroiliac joint |
| M0836 | Juvenile polyarthritis (seronegative) Lower leg, fibula, tibia, knee joint |
| M0837 | Juvenile polyarthritis (seronegative) Ankle and foot, metatarsus, tarsus, toes, ankle joint, other joints in foot |
| M0838 | Juvenile polyarthritis (seronegative) Other, head, neck, ribs, skull, trunk, vertebral column |
| M0839 | Juvenile polyarthritis (seronegative), site unspecified |
| M0840 | Pauciarticular juvenile arthritis, multiple sites |
| M0841 | Pauciarticular juvenile arthritis Shoulder region, clavicle, scapula, acromioclavicular, glenohumeral, sternoclavicular, (joints) |
| M0842 | Pauciarticular juvenile arthritis Upper arm, humerus, elbow joint |
| M0843 | Pauciarticular juvenile arthritis Forearm, radius, ulna, wrist joint |
| M0844 | Pauciarticular juvenile arthritis Hand, carpus, fingers, metacarpus, joints between these bones |
| M0845 | Pauciarticular juvenile arthritis Pelvic region and thigh, buttock, femur, pelvis, hip (joint), sacroiliac joint |
| M0846 | Pauciarticular juvenile arthritis Lower leg, fibula, tibia, knee joint |
| M0847 | Pauciarticular juvenile arthritis Ankle and foot, metatarsus, tarsus, toes, ankle joint, other joints in foot |
| M0849 | Pauciarticular juvenile arthritis, site unspecified |
| M0880 | Other juvenile arthritis : multiple sites |
| M0881 | Other juvenile arthritis Shoulder region, clavicle, scapula, acromioclavicular, glenohumeral, sternoclavicular, (joints) |
| M0882 | Other juvenile arthritis Upper arm, humerus, elbow joint |
| M0883 | Other juvenile arthritis Forearm, radius, ulna, wrist joint |
| M0884 | Other juvenile arthritis Hand, carpus, fingers, metacarpus, joints between these bones |
| M0885 | Other juvenile arthritis Pelvic region and thigh, buttock, femur, pelvis, hip (joint), sacroiliac joint |
| M0886 | Other juvenile arthritis Lower leg, fibula, tibia, knee joint |
| M0887 | Other juvenile arthritis Ankle and foot, metatarsus, tarsus, toes, ankle joint, other joints in foot |
| M0888 | Other juvenile arthritis Other, head, neck, ribs, skull, trunk, vertebral column |
| M0889 | Other juvenile arthritis : site unspecified |
| M0890 | Juvenile arthritis, unspecified : multiple sites |
| M0891 | Juvenile arthritis, unspecified Shoulder region, clavicle, scapula, acromioclavicular, glenohumeral, sternoclavicular, (joints) |
| M0892 | Juvenile arthritis, unspecified Upper arm, humerus, elbow joint |
| M0893 | Juvenile arthritis, unspecified Forearm, radius, ulna, wrist joint |
| M0894 | Juvenile arthritis, unspecified Hand, carpus, fingers, metacarpus, joints between these bones |
| M0895 | Juvenile arthritis, unspecified Pelvic region and thigh, buttock, femur, pelvis, hip (joint), sacroiliac joint |
| M0896 | Juvenile arthritis, unspecified Lower leg, fibula, tibia, knee joint |
| M0897 | Juvenile arthritis, unspecified Ankle and foot, metatarsus, tarsus, toes, ankle joint, other joints in foot |
| M0898 | Juvenile arthritis, unspecified Other, head, neck, ribs, skull, trunk, vertebral column |
| M0899 | Juvenile arthritis, unspecified : site unspecified |
| M0900 | Juvenile arthritis in psoriasis (L40.5+) : multiple sites |
| M0904 | Juvenile arthritis in psoriasis (L40.5+) Hand, carpus, fingers, metacarpus, joints between these bones |
| M0909 | Juvenile arthritis in psoriasis (L40.5+) : site unspecified |
| M0910 | Juvenile arthritis in Crohn's disease [regional enteritis] (K50.-+) : multiple sites |
| M0915 | Juvenile arthritis in Crohn's disease [regional enteritis] (K50.- +) Pelvic region and thigh, buttock, femur, pelvis, hip (joint), sacroiliac joint |
| M0919 | Juvenile arthritis in Crohn's disease [regional enteritis] (K50.-+) : site unspecified |
| M0924 | Juvenile arthritis in ulcerative colitis (K51.- +) Hand, carpus, fingers, metacarpus, joints between these bones |
| M0929 | Juvenile arthritis in ulcerative colitis (K51.-+) : site unspecified |
| M0980 | Juvenile arthritis in other diseases classified elsewhere : multiple sites |
| M0981 | Juvenile arthritis in other diseases classified elsewhere Shoulder region, clavicle, scapula, acromioclavicular, glenohumeral, sternoclavicular, (joints) |
| M0982 | Juvenile arthritis in other diseases classified elsewhere Upper arm, humerus, elbow joint |
| M0983 | Juvenile arthritis in other diseases classified elsewhere Forearm, radius, ulna, wrist joint |
| M0984 | Juvenile arthritis in other diseases classified elsewhere Hand, carpus, fingers, metacarpus, joints between these bones |
| M0985 | Juvenile arthritis in other diseases classified elsewhere Pelvic region and thigh, buttock, femur, pelvis, hip (joint), sacroiliac joint |
| M0986 | Juvenile arthritis in other diseases classified elsewhere Lower leg, fibula, tibia, knee joint |
| M0987 | Juvenile arthritis in other diseases classified elsewhere Ankle and foot, metatarsus, tarsus, toes, ankle joint, other joints in foot |
| M0988 | Juvenile arthritis in other diseases classified elsewhere Other, head, neck, ribs, skull, trunk, vertebral column |
| M0989 | Juvenile arthritis in other diseases classified elsewhere : site unspecified |
| M300 | Polyarteritis nodosa |
| M302 | Juvenile polyarteritis |
| M303 | Mucocutaneous lymph node syndrome [Kawasaki] |
| M308 | Other conditions related to polyarteritis nodosa |
| M310 | Hypersensitivity angiitis |
| M311 | Thrombotic microangiopathy |
| M313 | Wegener's granulomatosis |
| M314 | Aortic arch syndrome [Takayasu] |
| M317 | Microscopic polyangiitis |
| M318 | Other specified necrotizing vasculopathies |
| M319 | Necrotizing vasculopathy, unspecified |
| M320 | Drug-induced systemic lupus erythematosus |
| M321 | Systemic lupus erythematosus with involvement of organs and systems |
| M328 | Other forms of systemic lupus erythematosus |
| M329 | Systemic lupus erythematosus, unspecified |
| M350 | Sicca syndrome [Sjogren] |
| M351 | Other overlap syndromes |
| M352 | Behcet's disease |
| M357 | Hypermobility syndrome |
| M358 | Other specified systemic involvement of connective tissue |
| M359 | Systemic involvement of connective tissue, unspecified |
| M461 | Sacroiliitis, not elsewhere classified |
| M4622 | Osteomyelitis of vertebra : cervical region |
| M4624 | Osteomyelitis of vertebra : thoracic region |
| M4629 | Osteomyelitis of vertebra : site unspecified |
| M4640 | Discitis, unspecified : multiple sites in spine |
| M4646 | Discitis, unspecified : lumbar region |
| M4647 | Discitis, unspecified : lumbosacral region |
| M4649 | Discitis, unspecified : site unspecified |
| M4658 | Other infective spondylopathies : sacral and sacrococcygeal region |
| M4694 | Inflammatory spondylopathy, unspecified : thoracic region |
| M4696 | Inflammatory spondylopathy, unspecified : lumbar region |
| M8602 | Acute haematogenous osteomyelitis Upper arm, humerus, elbow joint |
| M8605 | Acute haematogenous osteomyelitis Pelvic region and thigh, buttock, femur, pelvis, hip (joint), sacroiliac joint |
| M8606 | Acute haematogenous osteomyelitis Lower leg, fibula, tibia, knee joint |
| M8607 | Acute haematogenous osteomyelitis Ankle and foot, metatarsus, tarsus, toes, ankle joint, other joints in foot |
| M861 | Other acute osteomyelitis |
| M8611 | Other acute osteomyelitis Shoulder region, clavicle, scapula, acromioclavicular, glenohumeral, sternoclavicular, (joints) |
| M8615 | Other acute osteomyelitis Pelvic region and thigh, buttock, femur, pelvis, hip (joint), sacroiliac joint |
| M8616 | Other acute osteomyelitis Lower leg, fibula, tibia, knee joint |
| M8617 | Other acute osteomyelitis Ankle and foot, metatarsus, tarsus, toes, ankle joint, other joints in foot |
| M8618 | Other acute osteomyelitis Other, head, neck, ribs, skull, trunk, vertebral column |
| M8619 | Other acute osteomyelitis : site unspecified |
| M8625 | Subacute osteomyelitis Pelvic region and thigh, buttock, femur, pelvis, hip (joint), sacroiliac joint |
| M8630 | Chronic multifocal osteomyelitis : multiple sites |
| M8635 | Chronic multifocal osteomyelitis Pelvic region and thigh, buttock, femur, pelvis, hip (joint), sacroiliac joint |
| M8636 | Chronic multifocal osteomyelitis Lower leg, fibula, tibia, knee joint |
| M8637 | Chronic multifocal osteomyelitis Ankle and foot, metatarsus, tarsus, toes, ankle joint, other joints in foot |
| M8638 | Chronic multifocal osteomyelitis Other, head, neck, ribs, skull, trunk, vertebral column |
| M8639 | Chronic multifocal osteomyelitis : site unspecified |
| M8647 | Chronic osteomyelitis with draining sinus Ankle and foot, metatarsus, tarsus, toes, ankle joint, other joints in foot |
| M8650 | Other chronic haematogenous osteomyelitis : multiple sites |
| M866 | Other chronic osteomyelitis |
| M8660 | Other chronic osteomyelitis : multiple sites |
| M8662 | Other chronic osteomyelitis Upper arm, humerus, elbow joint |
| M8663 | Other chronic osteomyelitis Forearm, radius, ulna, wrist joint |
| M8665 | Other chronic osteomyelitis Pelvic region and thigh, buttock, femur, pelvis, hip (joint), sacroiliac joint |
| M8666 | Other chronic osteomyelitis Lower leg, fibula, tibia, knee joint |
| M8667 | Other chronic osteomyelitis Ankle and foot, metatarsus, tarsus, toes, ankle joint, other joints in foot |
| M8668 | Other chronic osteomyelitis Other, head, neck, ribs, skull, trunk, vertebral column |
| M8669 | Other chronic osteomyelitis : site unspecified |
| M868 | Other osteomyelitis |
| M8680 | Other osteomyelitis : multiple sites |
| M8682 | Other osteomyelitis Upper arm, humerus, elbow joint |
| M8686 | Other osteomyelitis Lower leg, fibula, tibia, knee joint |
| M8687 | Other osteomyelitis Ankle and foot, metatarsus, tarsus, toes, ankle joint, other joints in foot |
| M869 | Osteomyelitis, unspecified |
| M8690 | Osteomyelitis, unspecified : multiple sites |
| M8691 | Osteomyelitis, unspecified Shoulder region, clavicle, scapula, acromioclavicular, glenohumeral, sternoclavicular, (joints) |
| M8692 | Osteomyelitis, unspecified Upper arm, humerus, elbow joint |
| M8693 | Osteomyelitis, unspecified Forearm, radius, ulna, wrist joint |
| M8694 | Osteomyelitis, unspecified Hand, carpus, fingers, metacarpus, joints between these bones |
| M8695 | Osteomyelitis, unspecified Pelvic region and thigh, buttock, femur, pelvis, hip (joint), sacroiliac joint |
| M8696 | Osteomyelitis, unspecified Lower leg, fibula, tibia, knee joint |
| M8697 | Osteomyelitis, unspecified Ankle and foot, metatarsus, tarsus, toes, ankle joint, other joints in foot |
| M8698 | Osteomyelitis, unspecified Other, head, neck, ribs, skull, trunk, vertebral column |
| M8699 | Osteomyelitis, unspecified : site unspecified |
| N39 | Other disorders of urinary system |
| N390 | Urinary tract infection, site not specified |
| T88 | Other complications of surgical and medical care, not elsewhere classified |
| T887 | Unspecified adverse effect of drug or medicament |

**Supplemental Table 2** Baseline characteristics between the development cohort and validation cohort.

| Parameters | Total  (n=318) | Development cohort (n=240) | Validation cohort (n=78) | *p*-value |
| --- | --- | --- | --- | --- |
| Age | 6.6 (3.6-11.3) | 6.4 (3.4-11.6) | 6.6 (4-10.3) | 0.837 |
| Female, n (%) | 154 (48.4) | 124 (51.7) | 30 (38.5) | 0.05 |
| Duration of fever | 19.5 (11-34) | 19 (11-34.8) | 20 (12-34) | 0.397 |
| Clinical manifestations, n (%) | | | | |
| Headache | 26 (8.2) | 20 (8.3) | 6 (7.7) | 0.858 |
| Cough | 77 (24.2) | 67 (27.9) | 10 (12.8) | 0.007* |
| Poor appetite | 104 (32.7) | 81 (33.8) | 23 (29.5) | 0.486 |
| Vomiting | 3 (23.1) | 3 (10) | 1 (2.9) | 0.073 |
| Abdominal pain | 1 (7.7) | 1 (3.3) | 2 (5.7) | 0.819 |
| Diarrhea | 3 (23.1) | 4 (13.3) | 2 (5.7) | 0.219 |
| Evanescent rash | 27 (11.3) | 27 (11.3) | 4 (5.1) | 0.113 |
| Photosensitivity | 6 (1.9) | 5 (2.1) | 1 (1.3) | 1.000 |
| Weight loss | 45 (14.2) | 34 (14.2) | 11 (14.1) | 0.989 |
| Malaise | 39 (12.3) | 35 (14.6) | 4 (5.1) | 0.027* |
| Refuse to walk | 27 (8.5) | 14 (5.8) | 13 (16.7) | 0.003* |
| Fatigue | 22 (6.9) | 12 (5) | 10 (12.8) | 0.018* |
| Dyspnea | 19 (6) | 12 (5) | 7 (9) | 0.268 |
| Pallor | 147 (46.2) | 112 (46.7) | 35 (44.9) | 0.782 |
| Hepatomegaly | 154 (48.4) | 115 (47.9) | 39 (50) | 0.749 |
| Splenomegaly | 94 (29.6) | 73 (30.4) | 21 (26.9) | 0.557 |
| Arthritis | 88 (27.7) | 58 (24.2) | 30 (38.5) | 0.014* |
| Lymphadenopathy | 135 (42.5) | 107 (44.6) | 28 (35.9) | 0.178 |
| Petechiae | 35 (11) | 25 (10.4) | 10 (12.8) | 0.556 |
| Rash | 58 (18.2) | 49 (20.4) | 9 (11.5) | 0.078 |
| Malar rash | 10 (3.1) | 9 (3.8) | 1 (1.3) | 0.461 |
| Discoid rash | 10 (3.1) | 10 (4.2) | 0 (0) | - |
| Oral ulcer | 17 (5.3) | 17 (7.1) | 0 (0) | - |
| Laboratory data | | | | |
| Hb (g/dL) | 9.4 (7.6-10.9) | 9.5 (7.9-11) | 9.1 (7.4-10.5) | 0.264 |
| Hct (%) | 29.3 (23.8-33.1) | 29.5 (24.5-33.4) | 29.2 (22.2-32.3) | 0.208 |
| WBC (cells/mm^3^) | 11,075  (6,050-18,360) | 10,955  (6,032.5-18,360) | 11,120 (6,040-18,600) | 0.931 |
| ANC (cells/mm^3^) | 5,007.1  (1,572.4-10,239.6) | 5,241.7  (1,961.6-10,692.2) | 3,487.4  (600-9,298.8) | 0.079 |
| ALC (cells/mm^3^) | 2,538.2  (1,448.8-4,463.7) | 2,612  (1,454.4-4,565.6) | 2,388.1  (1,694-4,137.6) | 0.580 |
| Platelets (cells/mm^3^) | 273,000  (119,500-430,750) | 284,500  (133,250-430,750) | 224,000  (57,000-419,000) | 0.164 |

Data presented as median (percentile 25th-percentile 75th). **p-*value < 0.05 was set as significance. Hb, hemoglobin; Hct, hematocrit; WBC, white blood cell count; ANC, absolute neutrophil count; ALC, absolute lymphocyte count.
